# The Distinct Conformational Dynamics of K-Ras and H-Ras A59G

**DOI:** 10.1371/journal.pcbi.1000922

**Published:** 2010-09-09

**Authors:** Suryani Lukman, Barry J. Grant, Alemayehu A. Gorfe, Guy H. Grant, J. Andrew McCammon

**Affiliations:** 1 Department of Chemistry, University of Cambridge, Cambridge, United Kingdom; 2 Bioinformatics Institute, Agency for Science, Technology and Research, Singapore; 3 Department of Chemistry and Biochemistry, Center for Theoretical Biological Physics, University of California San Diego, La Jolla, California, United States of America; 4 Howard Hughes Medical Institute, University of California San Diego, La Jolla, California, United States of America; 5 Department of Integrative Biology and Pharmacology, University of Texas Health Science Center, Houston, Texas, United States of America; 6 Department of Pharmacology, University of California San Diego, La Jolla, California, United States of America; University of Houston, United States of America

## Abstract

Ras proteins regulate signaling cascades crucial for cell proliferation and differentiation by switching between GTP- and GDP-bound conformations. Distinct Ras isoforms have unique physiological functions with individual isoforms associated with different cancers and developmental diseases. Given the small structural differences among isoforms and mutants, it is currently unclear how these functional differences and aberrant properties arise. Here we investigate whether the subtle differences among isoforms and mutants are associated with detectable dynamical differences. Extensive molecular dynamics simulations reveal that wild-type K-Ras and mutant H-Ras A59G are intrinsically more dynamic than wild-type H-Ras. The crucial switch 1 and switch 2 regions along with loop 3, helix 3, and loop 7 contribute to this enhanced flexibility. Removing the gamma-phosphate of the bound GTP from the structure of A59G led to a spontaneous GTP-to-GDP conformational transition in a 20-ns unbiased simulation. The switch 1 and 2 regions exhibit enhanced flexibility and correlated motion when compared to non-transitioning wild-type H-Ras over a similar timeframe. Correlated motions between loop 3 and helix 5 of wild-type H-Ras are absent in the mutant A59G reflecting the enhanced dynamics of the loop 3 region. Taken together with earlier findings, these results suggest the existence of a lower energetic barrier between GTP and GDP states of the mutant. Molecular dynamics simulations combined with principal component analysis of available Ras crystallographic structures can be used to discriminate ligand- and sequence-based dynamic perturbations with potential functional implications. Furthermore, the identification of specific conformations associated with distinct Ras isoforms and mutants provides useful information for efforts that attempt to selectively interfere with the aberrant functions of these species.

## Author Summary

The proto-oncogene Ras mediates signaling pathways controlling cell proliferation and development by cycling between active and inactive conformational states. Mutations that affect the ability to switch between states are associated with over 25% of human tumors. However, despite much effort, details of how these mutations affect the fidelity of activating conformational transitions remain unclear. Here we employ extensive molecular dynamics simulations combined with principal component analysis to investigate whether the subtle differences among functionally distinct isoforms and oncogenic mutants are associated with detectable dynamical differences. Our results reveal that wild-type K-Ras, the most prevalent isoform in a number of cancers, and mutant H-Ras A59G are intrinsically more dynamic than wild-type H-Ras. Furthermore, we have observed the first spontaneous GTP-to-GDP transition of H-Ras A59G during unbiased molecular dynamics simulation. These results indicate that key changes in sequence can lead to different dynamic properties that may be relevant for the unique physiological and aberrant functions of Ras isoforms and mutants. Furthermore, the current results shed further light on the conformational transition mechanism of this important molecular switch.

## Introduction

Ras proteins couple cell-surface receptors to intracellular signaling cascades involved in cell proliferation, differentiation and development. Signal propagation through Ras is mediated by a regulated GTPase cycle that leads to active and inactive conformations with distinct affinity for downstream effectors. Regulatory proteins including guanine nucleotide exchange factors (GEFs) and GTPase-activating proteins (GAPs) stimulate the intrinsically slow GTPase cycle promoting proper signal flow. Ras mutants with an impaired GTPase activity that are insensitive to the action of GAPs and GEFs result in prolonged downstream signaling associated with oncogenic cell growth in diverse human cancers and leukemia [1], [2].

Ras genes encode multiple isoforms of which H-, N-, and K-Ras are the most abundant. K-Ras can be found as two splice variants termed K-Ras4A and K-Ras4B. Although these isoforms share a high degree of similarity (over 90% sequence identity), their physiological functions are not necessarily equivalent [3]–[9]. K-Ras4B is essential for normal mouse embryogenesis and development, whereas H-, N-, and K-Ras4A are dispensable when K-Ras4B is present [4], [5]. K-Ras plays a unique role in cardiovascular homeostasis as mutant mice with their K-Ras gene modified to encode H-Ras, exhibit dilated cardiomyopathy associated with arterial hypertension[10]. Furthermore, mutations in K-Ras occur most commonly in human cancers and developmental diseases, including pancreatic, colorectal, lung, cervical and hematological cancer, Noonan syndrome, and Cardio-facio-cutaneous syndrome [11]–[13].

**Figure 1:**
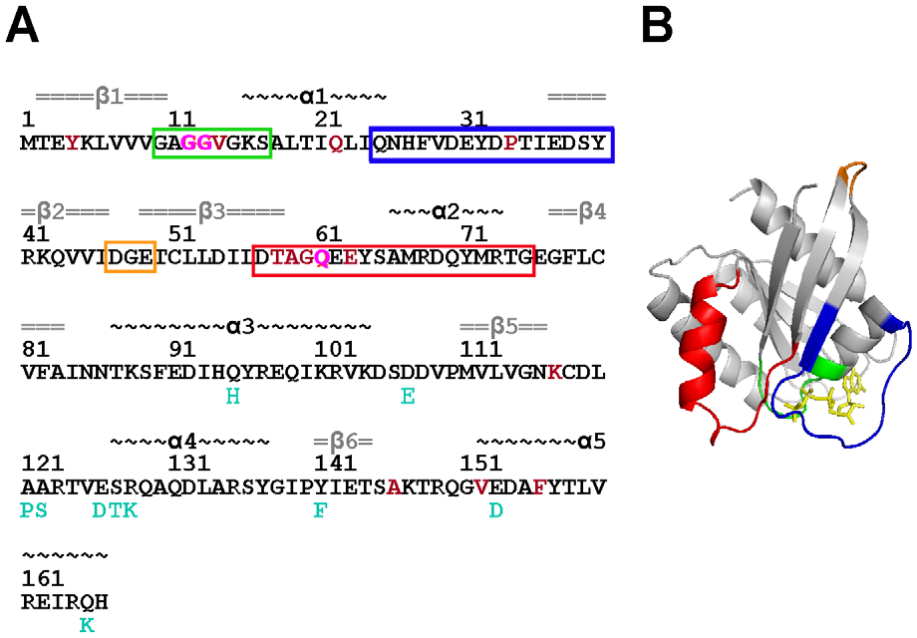
The primary and tertiary structures of Ras. (A) The sequence of H-Ras and K-Ras (amino acid differences between K-Ras and H-Ras are indicated in cyan). Residues 12, 13, and 61, whose substitutions are associated with a large number of cancers, are colored magenta. Other residues where mutations have been reported associated with various cancers and developmental diseases, are colored in brown. Secondary structure content is indicated on top of amino acid positions with α-helices (black) and β-sheets (gray) (B) The Ras catalytic domain is composed of a six-stranded central β-sheet surrounded by five α-helices. Nucleotide binds to a conserved phosphate-binding loop (P-loop comprising residues 10–17, green) and two switch loop regions (switch 1, residues 25–40, blue, and switch 2, residues 57–75, red). Oncogenic mutations often occur at these regions, particularly residues 12, 13 and 61 [12]. The loop 3 region (orange) is also highlighted.

The unique functions of Ras isoforms are mediated by their preferences for different binding partners [14]. Thus an understanding of functional fidelity requires a detailed structural and dynamical characterization of each isoform. The recently solved atomic structure of K-Ras4B, hereafter referred as K-Ras, revealed a high degree of similarity to previously solved H-Ras structures (94% sequence identity and 1.03 Å Cα RMSD over the 166 residues of the catalytic domain) (Fig 1). Given these small differences it is currently unclear how distinct binding preferences might arise.

Small changes in sequence can lead to different dynamic properties, which are manifested as only subtle changes in the average structure observed by x-ray crystallography. Recently we reported the observation of spontaneous nucleotide-dependent transition during unbiased molecular dynamics (MD) simulation of the oncogenically active H-Ras G12V variant [15]. This study suggested the existence of a lower thermally accessible energetic barrier between inactive and active states of this variant that renders it prone to adopt an active conformational state. This dynamic effect is not apparent from comparing the crystallographic structures of H-Ras G12V to wild-type H-Ras. Since such a single residue substitution on H-Ras could have dramatic effects on its dynamics, we hypothesize that a few residue substitutions among isoforms might influence their structural dynamics and hence their preferences and affinities to ligands and binding partners.

Previous classical and accelerated MD simulations of H-Ras have successfully characterized its dynamic features and proposed a reaction path for the ligand-associated conformational changes [16]–[18]. Furthermore, classical MD simulations of homology-built K-and N-Ras in the nucleotide-free state have suggested enhanced flexibility of K-Ras relative to other isoforms [15]. However, it is important to determine if this conclusion holds also in the presence of the nucleotide and is not affected by the initial structure. Since downstream effectors bind to active, GTP-bound Ras, the first part of our present study has been focused on investigating the dynamics of GTP-bound Ras isoforms.

Here we employ multi-copy MD simulations to investigate whether the subtle differences between H- and K-Ras isoforms are associated with detectable dynamical differences that might have potential functional consequences. We first conducted an expanded bioinformatic analysis of available Ras crystallographic structures and found that K-Ras has similar conformational features to the H-Ras A59G mutant. We further performed MD simulations on active, GTP-bound wild-type K-Ras, H-Ras, and H-Ras A59G. Indeed, we observed that both the wild-type K-Ras and H-Ras A59G variant are similarly more dynamic than wild-type H-Ras. However, wild-type K-Ras also has dynamic features that are similar to those of wild-type H-Ras, hence wild-type functions are preserved. The different dynamic features between wild-type K- and H-Ras may also provide clues on distinct preferences for binding proteins and hence subsequent downstream signaling functions. Another major contribution of this study is the first report on the spontaneous GTP-to-GDP transition of the H-Ras A59G variant. The H-Ras A59G variant crystallized with a GTP-analog, when simulated with a GDP, is capable of spontaneously adopting GDP-bound conformations within 20 ns. The atomic details of this transition are important for understanding Ras signaling and function.

## Results/Discussion

We first examined the structure of K-Ras in relation to other available Ras experimental structures. This analysis revealed a similarity to a A59G H-Ras variant. We then performed multiple MD simulations on wild-type K-, H-Ras, and H-Ras A59G. The Ras isoforms exhibited differences in their active, GTP-bound conformational dynamics. H-Ras A59G variant simulated with GDP was able to achieve a spontaneous GTP-to-GDP transition.

### The K-Ras crystal structure is similar in conformation to A59G H-Ras structure

**Figure 2:**
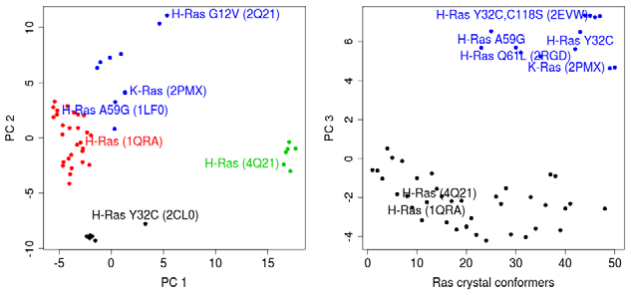
Results of principal component analysis (PCA) on the Ras catalytic domains reveal the relationship between K-Ras and other H-Ras crystal structures. Structures are projected onto the first three principal components (PC) with the largest variances and colored based on the dominant grouping obtained from hierarchical clustering of the projected structures. Representative structures corresponding to distinct bound-nucleotides, wild-type or mutant states, and different isoforms are labeled with their PDB IDs.

To investigate the relationship of K-Ras to other available Ras structures, we compiled a crystallographic ensemble comprising 51 chains from the 47 unique Ras structures in the RCSB PDB [19], [20]. Principal component analysis (PCA) was used to characterize inter-conformer relationships. Despite the inclusion of ten new chains from six recently-solved structures, we obtained a similar distribution of conformers along the dominant principal components (PCs) as obtained in an earlier study [15]. This indicates the robustness of the distribution to the inclusion of new structures and the suitability of the method for describing new inter-conformer relationships. Two major clusters are again evident along PC1 corresponding to distinct GTP and GDP bound conformations (Fig 2), confirming the previous observation that different chemical molecules at the active sites are associated with different global Ras conformations [15]. Note that some GTP/GTP-analog-bound structures were not situated within the main GTP-cluster. These structures have a mutation at the P-loop or switch regions. For example, H-Ras G12V, H-Ras A59G, and H-Ras Y32C (PDB: 2Q21, 1LF0, and 2CL0 respectively) did not reside in the GTP-cluster. Interestingly, the wild-type K-Ras conformer also resides outside the main GTP and GDP clusters in close proximity to the crystal conformer of H-Ras A59G mutant. Both structures are indeed similar in terms of Cα RMSD (0.68 Å).

While the first principal component revealed distinct clusters of Ras conformations corresponding to different bound-nucleotides (Fig 2), the third principal component showed distinct clusters of Ras conformations corresponding to mutant states (Fig 2). Ras crystal conformers with mutations at critical residues 32, 59, 61, and 118, affecting GTP hydrolysis, emerged far from the wild-type H-Ras conformers along the third principal component. Two H-Ras mutant conformers, G12P and G60A were found to reside within the majority wild-type cluster. Interestingly, these mutations are non-oncogenic. For example H-Ras G12P is known to possess wild-type like intrinsic GTP hydrolysis and GDP dissociation functions [21]. In summary, the current principal component projections based on the Ras crystallographic ensemble are robust to the inclusion of new structures and facilitate the discrimination of key ligand- and sequence-based structural perturbations.

### Wild-type K-Ras and H-Ras A59G are more dynamic than wild-type H-Ras

To further probe the conformational dynamics of the different Ras isoforms we performed multi-copy molecular dynamics simulations on the following systems: wild-type K-Ras, wild-type H-Ras, and H-Ras A59G. Although the starting wild-type K-Ras and the wild-type H-Ras structures are similar in overall conformation (Cα RMSD is 1.03 Å), the active site of wild-type K-Ras is more similar to that of H-Ras A59G (Cα RMSD is 1.16 Å) than wild-type H-Ras (Cα RMSD is 1.74 Å) (Fig S1).

**Figure 3:**
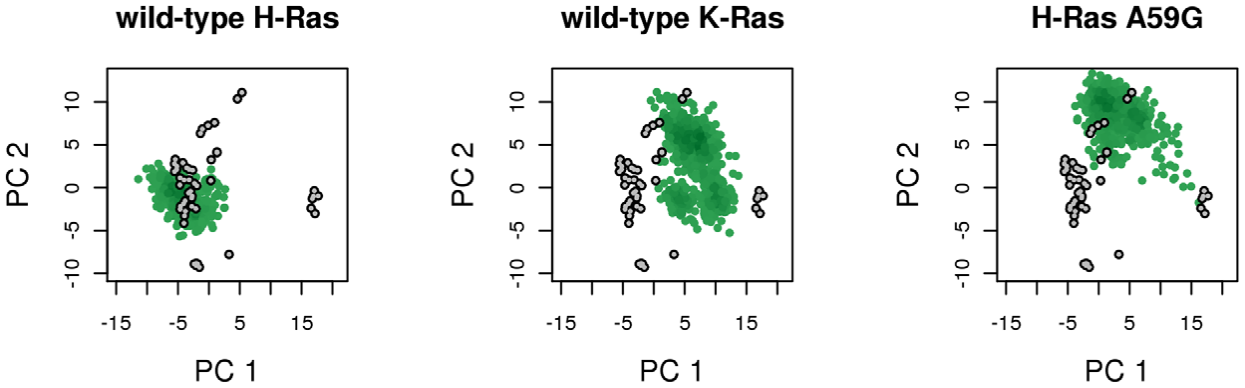
Projections of MD conformers onto the first and second principal components (PC) obtained from the analysis on the Ras crystallographic ensemble. The grey and green points represent crystallographic and MD conformers respectively.

**Figure 4:**
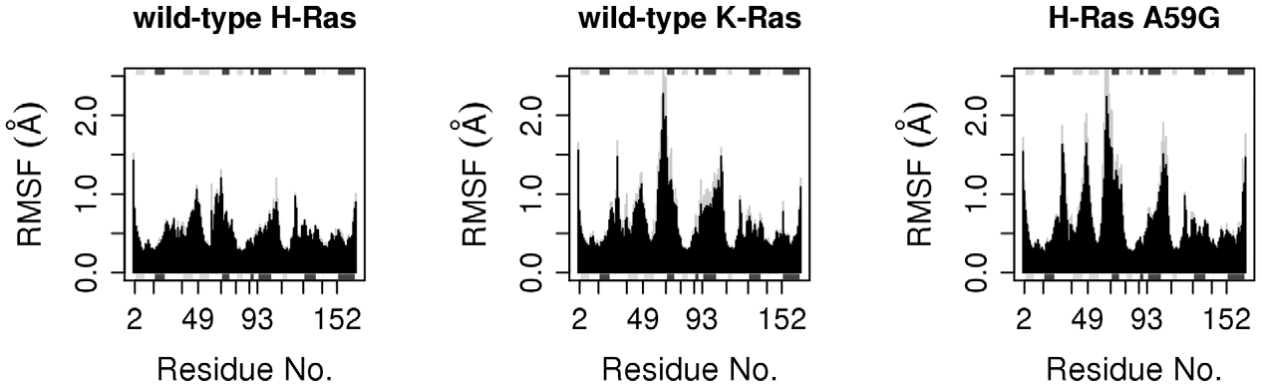
Average root mean square fluctuations (RMSF) of H-Ras, K-Ras, and H-Ras A59G from three sets of MD simulations for each system. The standard deviations about the average are shown in gray.

Comparison of the wild-type K-Ras and H-Ras trajectories in the GTP-bound form revealed variations in their dynamic behaviors, consistent with previous MD simulations of homology-built K-Ras and wild-type H-Ras in the nucleotide-free state [15]. Interestingly, wild-type K-Ras is similar to H-Ras A59G in sampling a wider region of conformational space proximal to the H-Ras G12V variant crystal conformer (Fig 3). Previous MD simulations of H-Ras G12V also displayed enhanced sampling when compared to wild-type H-Ras [15]. In terms of RMSD, the MD conformers of wild-type K-Ras and H-Ras A59G appear to be more similar to each other than to the MD conformers of wild-type H-Ras (Fig S2). The same trend is apparent for the canonical switches. For example, across the three sets of MD trajectories of the wild-type H-Ras, switch 1 RMSD values are generally higher than those of switch 2 (Fig S3) whereas the opposite is true for K-Ras and H-Ras A59G. Comparing RMSF values from the respective GTP-bound simulations indicates that wild-type K-Ras and H-Ras A59G are indeed more dynamic than wild-type H-Ras (Fig 4). These differences are significant as indicated by paired student's t tests (P<0.01). Particularly noteworthy are residues at the crucial switch 1, switch 2, loop3, α3 helix, and loop 7 regions, all of which are more flexible in K-Ras and A59G than in wild-type H-Ras.

### Isoforms have distinct correlated motions and active site configurations

**Figure 5:**
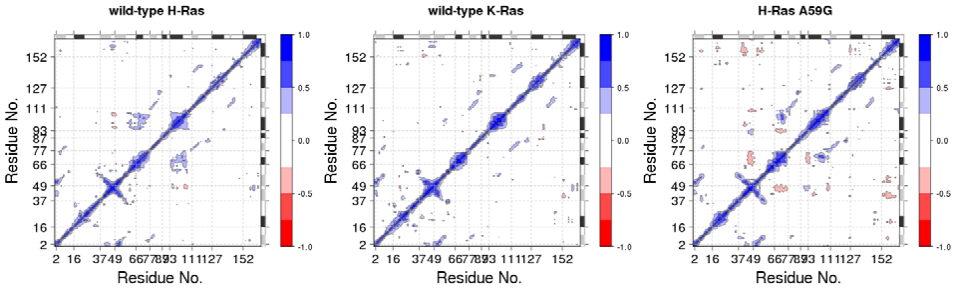
Dynamic cross-correlation maps (DCCMs) reveal the extent of correlation for all residue pairs in the three sets of MD simulations for the wild-type H-Ras, K-Ras, and H-Ras A59G. Only correlation coefficients with an absolute value greater than 0.25 are displayed. Motion occurring along the same direction is represented by positive correlation (blue), whereas anti-correlated motion occurring along the opposite direction is represented by negative correlation (red).

To identify regions undergoing correlated motions we analyzed dynamic cross-correlation maps for the three sets of trajectories. The correlated motion between loop 3 and α5 helix is absent in H-Ras A59G but present in the wild-type K- and H-Ras (Fig 5). This suggests that the substitution at position 59 in switch 2 affects the correlated motion of structural elements that are sequentially and spatially distant. The loop 3 and the C-terminal of α5 helix are parts of switch 3 [22] and correlated motion between loop 3 and α5 was reported in previous accelerated MD simulations of wild-type H-Ras [23]. On the other hand, wild-type H-Ras and H-Ras A59G exhibit a similar pattern of correlated motions between α2 and α3-loop 7 (Fig 5). The α3-loop 7 region has recently been reported to function as an allosteric site regulating switch 2 ordering [24].

**Figure 6:**
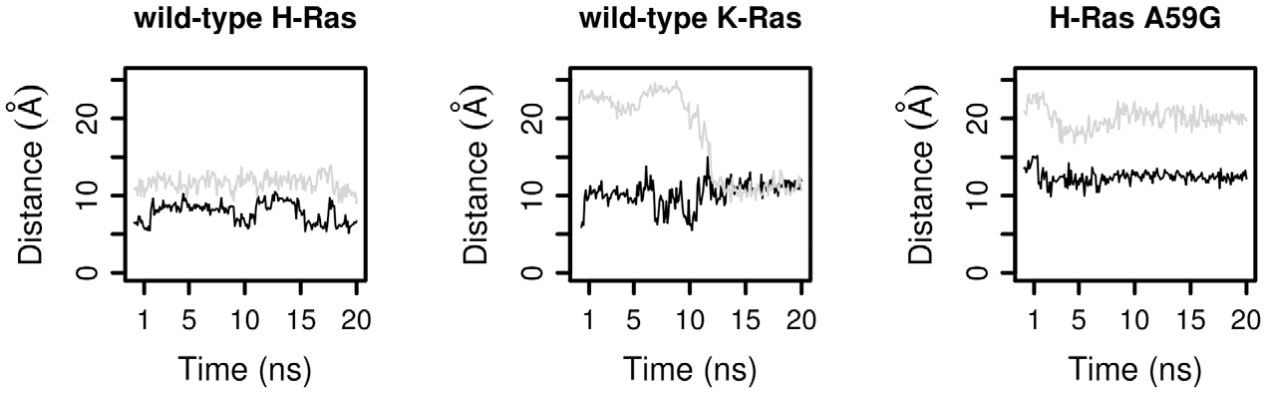
The distances between residue Q61 and γ-phosphate (black), between residue Y64 and γ-phosphate (gray) in the wild-type H-Ras, wild-type K-Ras, and H-Ras A59G.

Residue Q61 of switch 2 is essential for GTP hydrolysis with its side chain involved in orienting and activating a nucleophilic water molecule [25]. To monitor the conformation of Q61, we tracked the distances between the γ-phosphate and Q61 side chain atom NE2. Their distances are greater in wild-type K-Ras than in wild-type H-Ras, although not as large as in H-Ras A59G simulations (Fig 6). Such displacement of Q61 toward R68 and away from the catalytic site likely contributes to the much slower intrinsic GTP hydrolysis of H-Ras A59G when compared to wild-type H-Ras [26].

**Figure 7:**
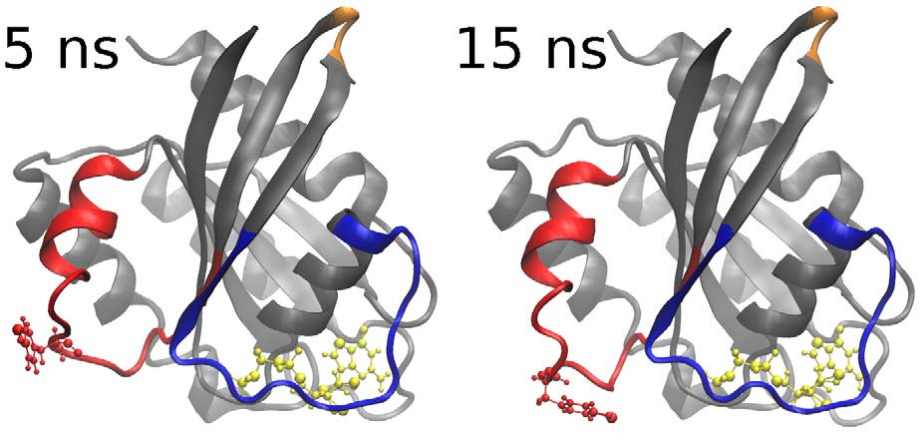
The wild-type K-Ras side chain of residue Y64 in switch 2 (red) changes orientation and moves closer toward the GTP throughout the MD simulation. The switch 2 of 15-ns K-Ras conformation resembles the switch 2 in the wild-type H-Ras crystallographic conformation.

Y64 is more distant to the γ-phosphate in both wild-type K-Ras and H-Ras A59G than in wild-type H-Ras (Fig 6). In simulations of wild-type H-Ras, the side chain of Y64 was persistently oriented and positioned close to the γ-phosphate of GTP (distance between Y64 OH atom and γ-phosphate ranged from 9.0 to 13.9 Å, with an average value of 11.7 Å). In wild-type K-Ras, the distance varied between 9.2 and 25.7 Å, with an average value of 22.4 Å. In A59G, this distance ranged from 19.8 to 25.4 Å, and the average was 22.1 Å. GTP hydrolysis can only occur in conformations with the Y64 side chain being close to the γ-phosphate. Though the average distance of Y64 side chain and γ-phosphate in wild-type K-Ras is as long as in H-Ras A59G, wild-type K-Ras can also adopt conformations in which their Y64:OH and γ-phosphate distance is as short as that of wild-type H-Ras. Fig 7 depicts the change in the orientation of K-Ras Y64, the shortening of Y64:OH - γ-phosphate distance, and the motion of α2 helix toward the nucleotide-binding site.

### Spontaneous GTP-to-GDP transition of H-Ras A59G

The crystallographic structure of H-Ras A59G, on which the current simulations are based, was proposed to represent the conformation of wild-type H-Ras following β/γ-phosphate bond breakage but before γ-phosphate dissociation [26]. This mutant was also shown to have an impaired intrinsic GTP hydrolysis activity [26]. When we replaced the original GTP-analog with GDP and performed unbiased 20-ns MD simulation, we observed a spontaneous GTP-to-GDP transition. This spontaneous GTP-to-GDP transition complements our previous characterization of the spontaneous GDP-to-GTP transition observed in the H-Ras G12V variant [15]. The GTP-to-GDP transition has been previously analyzed in some studies [18], [27], [28] that employed biased calculations, such as targeted MD, which introduces external constraints to drive the transition. Different solvation conditions of explicit [18] or implicit solvent [27], were also used. Our study provided new insight on the GTP-to-GDP transition based on unbiased MD simulation in explicit solvent, which better mimics natural physiological conditions [29].

**Figure 8:**
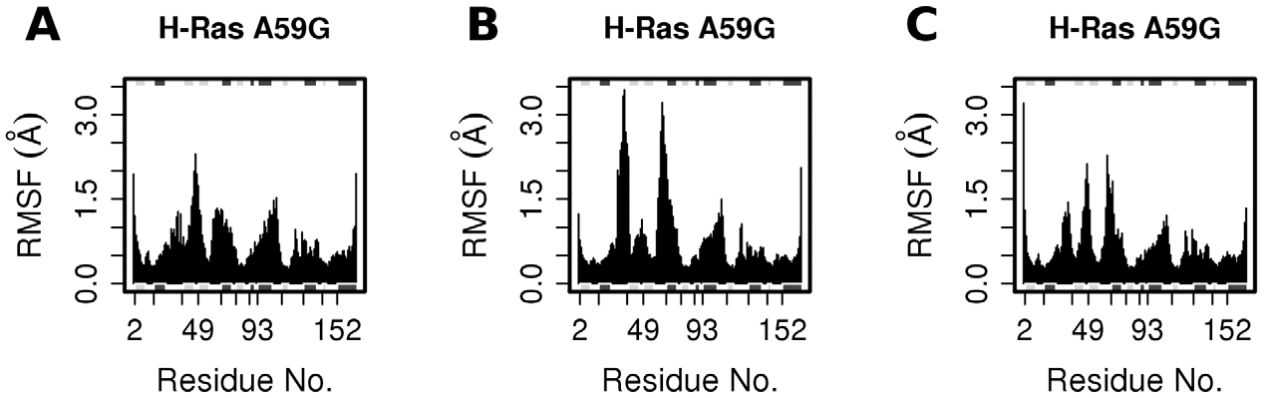
The RMSF of GDP-bound H-Ras A59G multiple MD simulations. (A) set 1, (B) 2, and (C) 3. Within 20-ns, only the set 2 simulation managed to reach the GDP-bound cluster of the Ras crystallographic ensemble.

We note that only one of our three multi-copy simulations managed to reach the GDP-cluster of the Ras crystallographic ensemble within 20-ns. Comparing the residue-wise flexibility from this simulation to the other two sets, indicates that the GTP-to-GDP transition involves higher RMSF at the loop 2, loop 4, and α2 regions, as well as lower RMSF at the loop 3 region (Fig 8). Below we focus on this transitioning in order to obtain atomic descriptions of the conformational conversion process.

**Figure 9:**
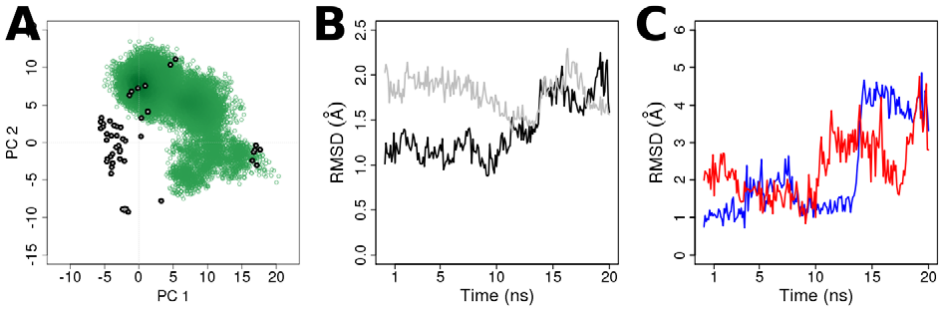
Spontaneous GTP-to-GDP transition in the GDP-bound H-Ras A59G MD simulation. (A) The projection of GDP-bound H-Ras A59G MD conformers onto the first two principal components of the crystallographic ensemble. (B) The RMSDs of H-Ras A59G MD conformers with respect to the GTP-bound H-Ras A59G crystal structure (PDB 1LF0, black) and the GDP-bound H-Ras A59G crystal structure (PDB 1LF5, gray). (C) The RMSD of switch 1 (blue) and switch 2 (red) during the MD simulation.

**Figure 10:**
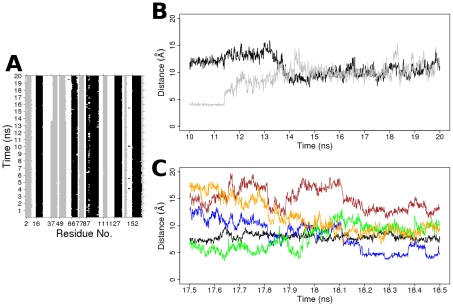
The atomic descriptions of spontaneous GTP-to-GDP transition in the H-Ras A59G MD simulation. (A) The dynamics of secondary structures of the GDP-bound H-Ras A59G MD conformers. (B) The distances between Y32-Y40 (black), Y32-β phosphate (gray). (C) The distances between G60 and β-phosphate (black), E37-R68 (blue), E37-Y64 (yellow), E37-Y71 (green), E37-Q61 (brown) between 17.5 and 18.5 ns of MD simulation.

When the MD-derived conformers are projected onto the first two principal components (PC) obtained from analyzing the Ras crystallographic ensemble, the GDP-bound H-Ras A59G MD conformers sample the GDP cluster of crystallographic conformers (PC 1: 15 to 20, PC 2: −5 to 0) (Fig 9A). Over time, the H-Ras A59G MD conformers evolve to resemble the GDP-bound more than the GTP-bound H-Ras A59G crystal structure (Fig 9B). Although both the GTP-to-GDP or GDP-to-GTP conformational changes involve rearrangements of the canonical switch regions [26] and partial unfolding and re-folding of helix 2, in each case multiple pathways are possible [27]. In the simulation, in the first 4 ns switch 2 has slightly higher RMSD values than switch 1 (Fig 9C) but the relative RMSD of the switches remains stable until 11.2 ns. Following this, the switch 2 RMSD increases significantly before the switch 1 RMSD catches up surpassing the switch 2 RMSD at about 13.8 ns. After a decrease in RMSD (∼16–18ns), the switches evolve again as the N-terminal of α2 helix unwinds (Fig 10A, Video S1). The unwinding of the α2 helical turn (residues 66–69) was also reported in a previous biased MD study of GTP-to-GDP conformational transitions [18]. Interestingly, despite some differences in the sequence of events, these results are similar to those reported based on the computation of minimum energy paths (MEPs) [27].

The side chain of Y32 lies across the nucleotide binding pocket in GTP-bound conformations but is displaced away from the binding pocket in GDP-bound H-Ras A59G and wild-type H-Ras crystal structures [21], [26], [30]. After 11.5 ns of simulation, Y32 moves away from nucleotide and comes closer to Y64 from 14 ns onwards (Fig 10B). The role of Y32 was also highlighted in the Y32F mutant with dysfunctional GTP hydrolysis [31]. Preceding the unwinding of helix 2 in switch 2, the Q61 of switch 2 orientates away from the switch 1 (Fig 10C). However, no significant change was seen in the distance between G60 and nucleotide preceding the unwinding of α2 (Fig 10C) as reported in [18]. This is to be expected as we commenced simulation form the A59G crystal structure conformation in which its G60, a possible hinge [32], had already moved away from the nucleotide to occupy the space made available by the A59G substitution. Next, the Y71 and R68 of switch 2 form close contacts with E37 of switch 1, stabilizing the protein during the α2 unwinding. The unwinding of α2 re-orientates switch 2 and brings Y64 closer to E37 of switch 1 (Fig 10C).

The MD conformers at different time frames also indicated interesting differences in their torsional angles with respect to the GTP-analog-bound versus the GDP-bound H-Ras A59G crystal conformers (Fig S4). The torsional angle differences were emphasized by the residues of loop 2 and loop 4 form switch 1 and 2 regions respectively. These regions exhibit correlated motions (Fig S5) and enhanced flexibility (Fig 8B) highlighting their roles in binding nucleotide and facilitating the GTP-to-GDP transition.

### The mechanisms of conformational change in Ras

Combining the current results with previous reports [15], [18], [23], [27] on the reaction paths for the transition between the active and inactive states of Ras, we arrive at the following conclusions. (i) Conformational changes accompanying GTP hydrolysis (GTP-to-GDP) and nucleotide exchange (GDP-to-GTP) involve common intermediates that are represented by the A59G structure; (ii) there is a barrier to reach this intermediate upon GTP hydrolysis, which is consistent with the 24.3 kcal/mol activation energy estimated by the minimum energy paths method for the GTP-to-GDP transition of wild-type H-Ras [27]; (iii) that an unbiased simulation of H-Ras A59G led to a transition from the GTP to the GDP state may suggest that this point mutation lowers the barrier between the intermediate and the GDP state; and (iv) once the large barrier between the GTP state and the H-Ras A59G-like intermediate is crossed, the remaining (multiple) barriers to the GDP-bound form can be crossed relatively easily. Finally, it is noteworthy that the PCA analysis enabled us to effectively cluster the diverse Ras crystal structures into two major clusters interspersed by intermediates that are predominantly populated by mutants. Some of these mutant structures are susceptible to nucleotide-associated transitions.

### Relevance for normal and aberrant Ras function

Oncogenic mutations may interfere with the intrinsic ability of Ras to hydrolyze GTP or lead to insensitivity to the action of GAPs. The loss or deregulation of intrinsic GTP hydrolysis seems to be sufficient for causing diseases [12], but GAP-insensitivity is still rescuable in some variants. For example, the H-Ras G12P and G12A variants are GAP-insensitive, but their intrinsic GTPase function and their ability to release GDP only modestly differ from those of wild-type [21], hence these mutants do not transform cells. The intrinsic GTP hydrolysis activity appears to be biologically sufficient to stop continuous downstream signaling [12], [33]. Therefore, structural changes that have the potential to affect the intrinsic GTPase function of Ras may also modulate its aberrant functions. Thus, the enhanced dynamics of K-Ras (and G59A H-ras) in functionally crucial regions may have an important implication for its normal and tumorigenic functions, which may partly explain the more frequent discovery of K-ras in cancer cells than any other Ras isoforms [34].

We speculate that the observed differences in dynamics may be related to the measured differences in the intrinsic GTPase activity of the three proteins. The intrinsic GTP hydrolysis rate of K-Ras (1.2×10^−4^ s^−1^ [33]) is roughly half the rate of the wild-type H-Ras (2.7×10^−4^ s^−1^ [24]) whereas that of H-Ras A59G (0.35×10^−4^ s^−1^ [26]) about eight times smaller. This may suggest that in the absence of GAP, K-Ras and H-Ras A59G spend more time in the GTP-bound conformational state than the wild-type H-Ras. The enhanced dynamics of K-Ras and H-Ras A59G may make the GTP-bound state entropically favored. This is consistent with a previous MD simulation on H-Ras G12V which revealed that the continuously changing active site makes GTP hydrolysis difficult [35]; the resulting elevation in the population of GTP-bound conformations will then lead to increased signaling through this oncogenic variant. However, caution is required in interpreting the existing rate constants as the measured intrinsic GTP hydrolysis rate is dependent on the GTP concentration present in the assay [25]. We thus believe that the current data would be even more useful if used in conjunction with GTP hydrolysis rates measured under identical experimental conditions.

### Conclusions

We have performed multiple unbiased MD simulation on wild-type K-Ras, H-Ras A59G and wild-type H-Ras. Results from these simulations support the observation that the active, GTP-bound wild-type K-Ras and H-Ras A59G variant are more dynamic than wild-type H-Ras. We observed spontaneous GTP-to-GDP transition during an unbiased MD simulation of H-Ras A59G. The approach of multivariate clustering of crystal structure conformations to reveal differences due to ligands and sequences highlights intermediate conformers, such as the H-Ras G12V [15] and A59G in this study, that facilitate faster sampling of large scale conformational transitions. We speculate that Ras variants which fall outside the major GTP- or GDP-clusters may be intrinsically more susceptible to transition than wild-type H-ras. Further systematic investigation of the structural and dynamic properties of Ras isoforms and mutants is likely to be informative for drug development, particularly drugs which selectively target distinct structural conformations associated with specific Ras isoforms or mutations. Structure based drug design efforts in this direction are currently underway.

## Materials and Methods

### Structure analysis

The Bio3D package [36] was used to retrieve homologous structures to K-Ras (PDB: 2PMX) from the PDB, perform principal component analysis (PCA) and additional trajectory analysis as described in [15], [23]. Core positions were first obtained from analysis of the crystallographic ensemble. Iterated rounds of structural superposition were used to identify the most structurally invariant region of the Ras structure. This procedure, implemented in the Bio3D package, entailed excluding those residues with the largest positional differences, before each round of superposition, until only the invariant “core” residues remained [36], [37]. The structurally invariant core was used as the reference frame for structural alignment of both crystal and simulation structures. Next, the Cartesian coordinates of aligned Cα atoms were used to define the elements of a covariance matrix. The covariance matrix was then diagonalized to derive principal components with their associated variances. After PCA, Ras crystal structures and MD conformers were projected on the subspace defined by principal components with the largest variances.

### Molecular dynamics (MD) simulation

Systems for molecular dynamics simulations were prepared from high-resolution crystal structures of wild-type K-Ras, wild-type H-Ras, and H-Ras A59G variant (PDB: 2PMX, 1QRA, and 1LF0 respectively). Each system was simulated with Mg^2+^GDP and Mg^2+^GTP. All simulations were performed with the AMBER 10 package [38]. The LEaP module was used for model construction, adding missing atoms to initial coordinates, and including parameters for guanine nucleotides [39] and Mg^2+^ ion. The protonation states for all titratable residues were determined using PDB2PQR [40]. The systems were neutralized using charge-neutralizing counter ions at pH 7, followed by explicit solvation with TIP3P water molecules with the buffering distance set to 10 Å. Energy minimization with decreasing constraints on the heavy atoms' positions, constant volume heating (to 300 K) was carried out for over 10 ps, followed by constant temperature (300 K) and constant pressure (1 atm) equilibration for additional 200 ps. The production simulation, using ff99SB force field [41], was conducted with time step of 2 fs, using the isobaric-isothermal ensemble at 300 K, 1 atm, and long-range non-bonded interactions with 10 Å atom-based cutoff. Electrostatic interactions were evaluated using the Particle-Mesh Ewald sum [42]. The SHAKE algorithm was used to constrain all covalent bonds involving hydrogen atoms. To enhance sampling and improve the statistical accuracy of the simulations, three independent MD simulations were performed on each system using different random initial velocities. Each simulation was performed for 20 ns resulting in 60 ns cumulative simulation time for each system.

## Supporting Information

Figure S1 Active site similarities of H-Ras (blue), K-Ras (black) and H-Ras A59G (red). The GTP nucleotide (yellow), switch 1 (residues 25–40) and switch 2 (residues 57–75) regions are displayed from the PDB entries 1QRA, 2PMX, and 1LF0 corresponding to H-, K- and H-Ras A59G respectively.
(0.39 MB TIF)

Figure S2 RMSD values of conformers obtained from three independent MD simulations with respect to Ras crystal structures. (A) The RMSD of wild-type K-Ras MD conformers with respect to the wild-type H-Ras (black) and H-Ras A59G (gray) crystal conformer. (B) The RMSD of H-Ras A59G MD conformers with respect to the wild-type H-Ras (black) and the wild-type K-Ras (gray) crystal conformer.
(0.34 MB TIF)

Figure S3 Root mean square deviations (RMSD) of switch 1 (residues 25–40, blue), switch 2 (residues 57–75, red), switch 3 (residues 47–49, 161–165, green) of H-Ras, K-Ras, and H-Ras A59G in three sets of MD trajectories of (A) wild-type H-Ras, (B) wild-type K-Ras, (C) H-Ras A59G.
(0.73 MB TIF)

Figure S4 Pseudo alpha Carbon torsion angle differences in MD conformers at different time point with respect to crystal structure of (A) GTP-analog-bound, (B) GDP-bound H-Ras A59G crystal conformers. Absolute values of the difference are plotted with secondary structures schematically depicted in black for helices and grey for strands.
(0.12 MB TIF)

Figure S5 The DCCM of GDP-bound H-Ras A59G MD simulation that achieved a spontaneous GTP-to-GDP transition.
(0.11 MB TIF)

Video S1 The GTP-to-GDP transition observed in the MD trajectory of H-Ras A59G. The N-terminal (residue 65–67) of α 2 helix unwinds. Important residues 37 (pink), 60 (black), 61 (red-brown), 64 (orange), 66 (magenta, to aid unwinding visualization), 68 (blue), and 71 (green), are highlighted in CPK style.URL: http://mccammon.ucsd.edu/~lukman/movie1_24May10.gif
(1.72 MB AVI)
