# Supplementary figures and images for: The Distinct Conformational Dynamics of K-Ras and H-Ras A59G
Source: PLoS Comput Biol. 2010 Sep 9;6(9):e1000922. doi: 10.1371/journal.pcbi.1000922 (PMC2936511; doi:10.1371/journal.pcbi.1000922)

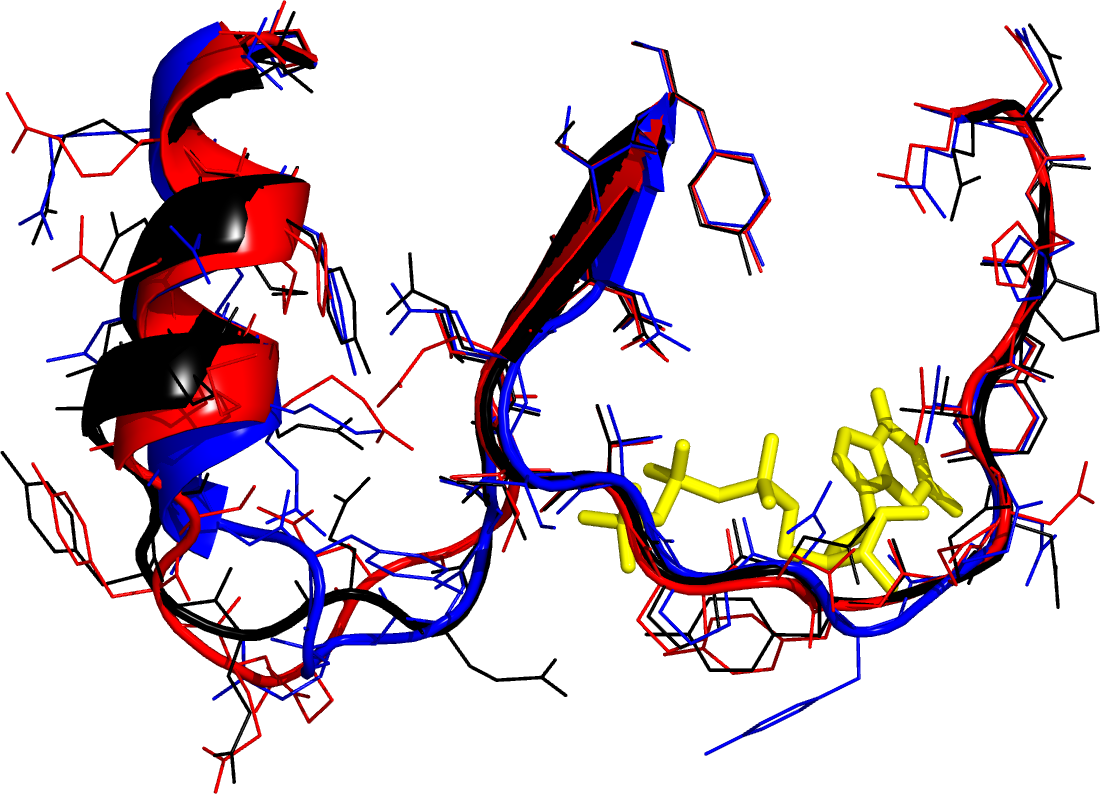

Supplement: Figure S1 — Active site similarities of H-Ras (blue), K-Ras (black) and H-Ras A59G (red). The GTP nucleotide (yellow), switch 1 (residues 25–40) and switch 2 (residues 57–75) regions are displayed from the PDB entries 1QRA, 2PMX, and 1LF0 corresponding to H-, K- and H-Ras A59G respectively. (0.39 MB TIF) [file pcbi.1000922.s001.tif]

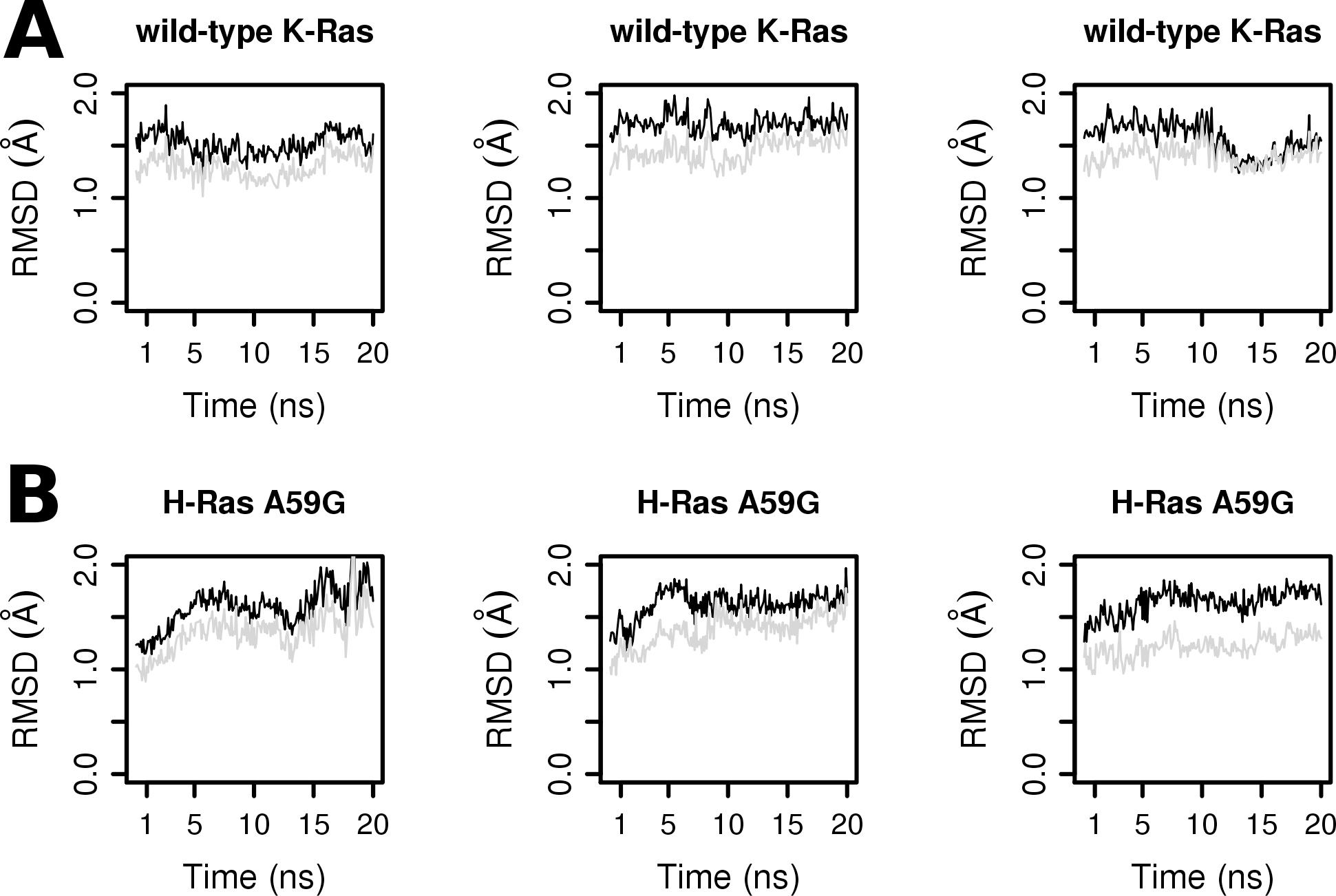

Supplement: Figure S2 — RMSD values of conformers obtained from three independent MD simulations with respect to Ras crystal structures. (A) The RMSD of wild-type K-Ras MD conformers with respect to the wild-type H-Ras (black) and H-Ras A59G (gray) crystal conformer. (B) The RMSD of H-Ras A59G MD conformers with respect to the wild-type H-Ras (black) and the wild-type K-Ras (gray) crystal conformer. (0.34 MB TIF) [file pcbi.1000922.s002.tif]

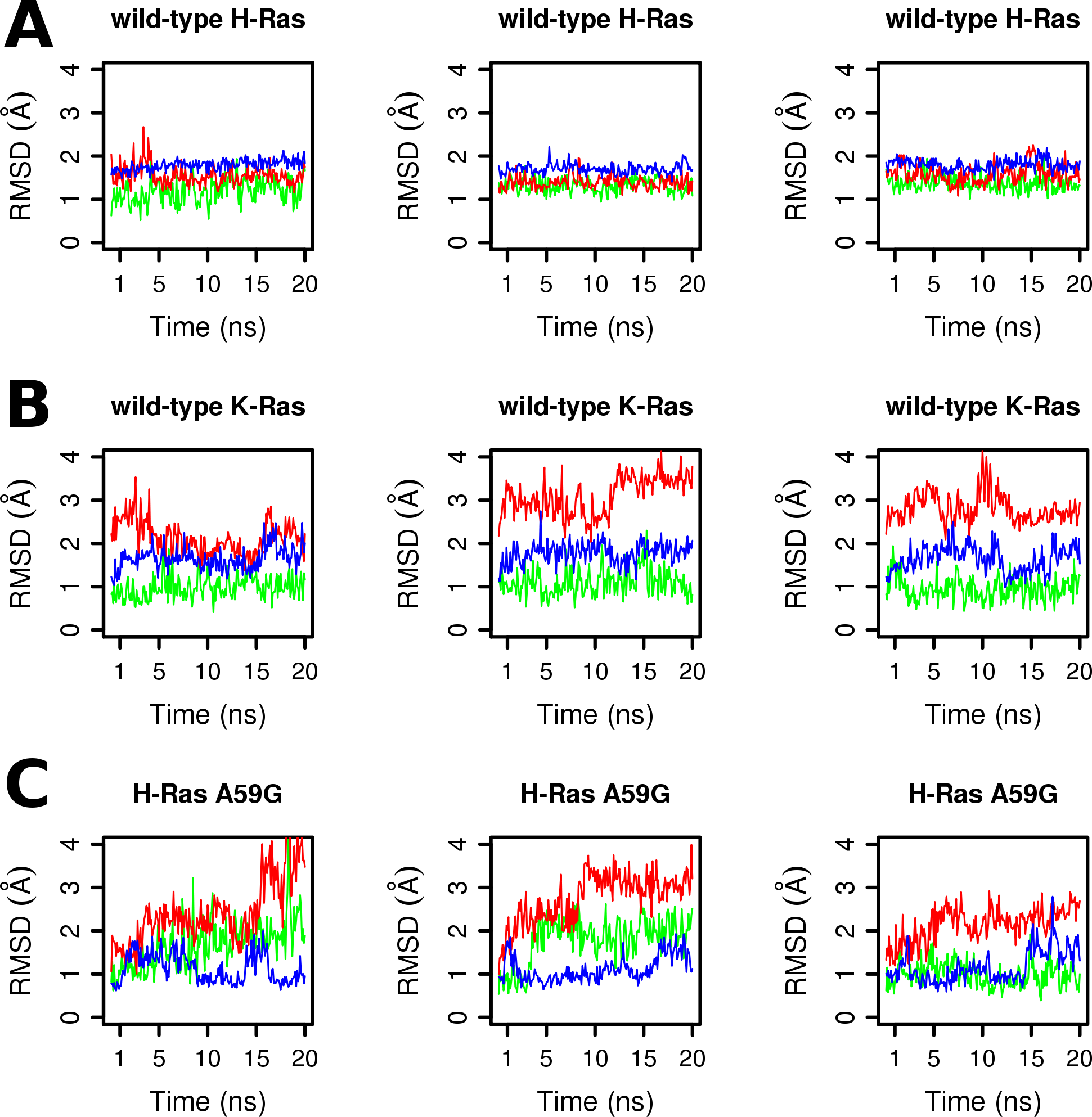

Supplement: Figure S3 — Root mean square deviations (RMSD) of switch 1 (residues 25–40, blue), switch 2 (residues 57–75, red), switch 3 (residues 47–49, 161–165, green) of H-Ras, K-Ras, and H-Ras A59G in three sets of MD trajectories of (A) wild-type H-Ras, (B) wild-type K-Ras, (C) H-Ras A59G. (0.73 MB TIF) [file pcbi.1000922.s003.tif]

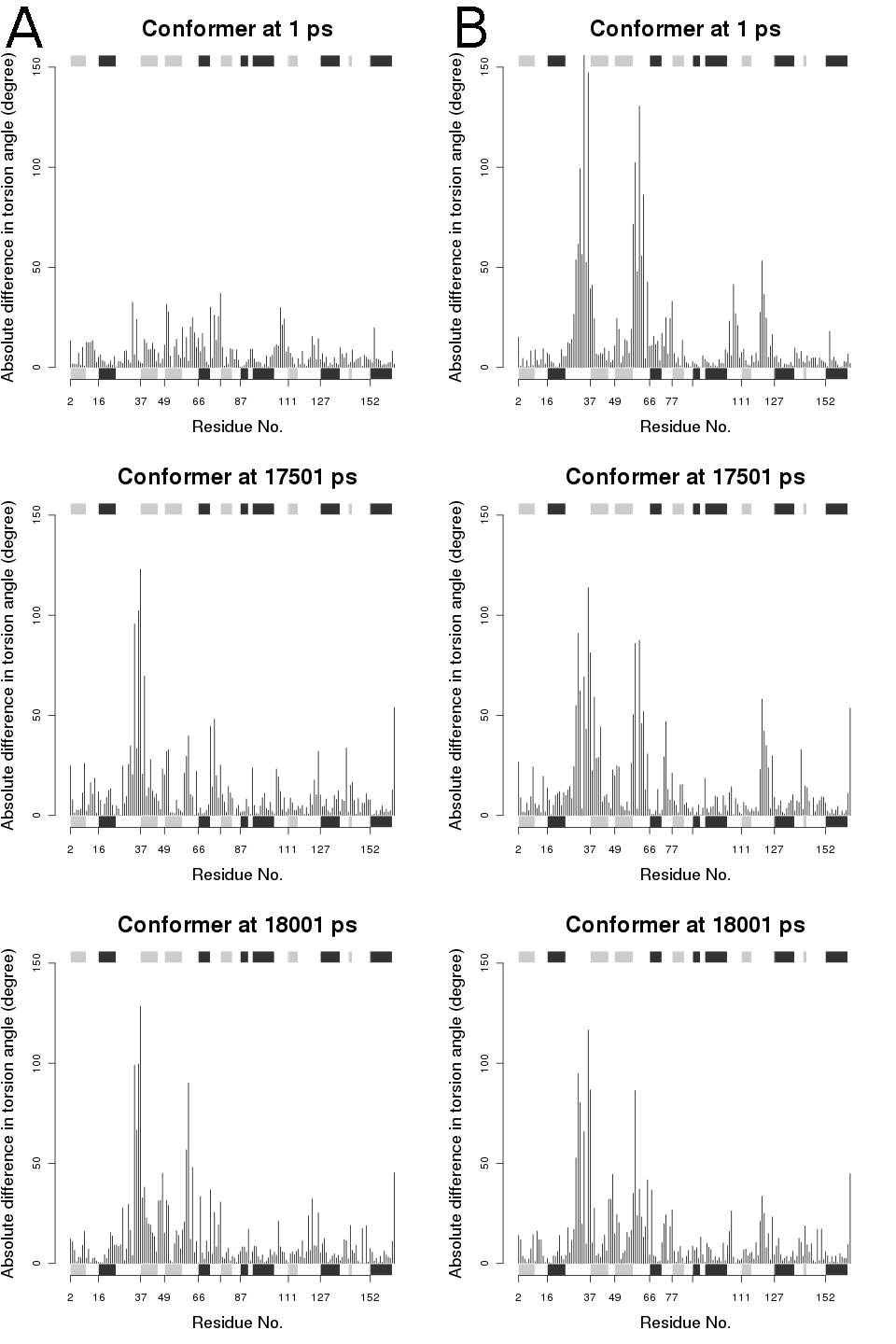

Supplement: Figure S4 — Pseudo alpha Carbon torsion angle differences in MD conformers at different time point with respect to crystal structure of (A) GTP-analog-bound, (B) GDP-bound H-Ras A59G crystal conformers. Absolute values of the difference are plotted with secondary structures schematically depicted in black for helices and grey for strands. (0.12 MB TIF) [file pcbi.1000922.s004.tif]

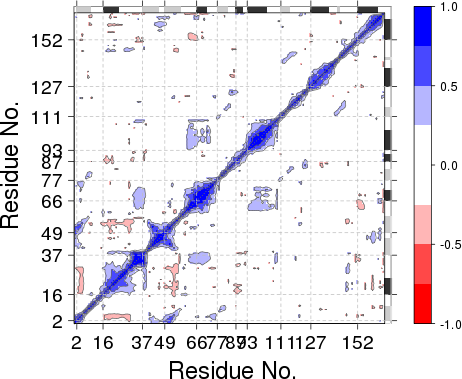

Supplement: Figure S5 — The DCCM of GDP-bound H-Ras A59G MD simulation that achieved a spontaneous GTP-to-GDP transition. (0.11 MB TIF) [file pcbi.1000922.s005.tif]
